# Hydrolytic Metallo-Nanozymes: From Micelles and Vesicles to Gold Nanoparticles

**DOI:** 10.3390/molecules21081014

**Published:** 2016-08-04

**Authors:** Fabrizio Mancin, Leonard J. Prins, Paolo Pengo, Lucia Pasquato, Paolo Tecilla, Paolo Scrimin

**Affiliations:** 1 Department of Chemical Sciences, University of Padova, via Marzolo, 1, Padova 35131, Italy; fabrizio.mancin@unipd.it (F.M.); leonard.prins@unipd.it (L.J.P.); 2 Department of Chemical and Pharmaceutical Sciences, University of Trieste, via Giorgieri, 1, Trieste 34127, Italy; ppengo@units.it (P.P.); lpasquato@units.it (L.P.); ptecilla@units.it (P.T.)

**Keywords:** micelles, vesicles, aggregation colloids, gold nanoparticles, phosphate cleavage, carboxylate cleavage, hydrolysis, Zn(II), Cu(II)

## Abstract

Although the term nanozymes was coined by us in 2004 to highlight the enzyme-like properties of gold nanoparticles passivated with a monolayer of Zn(II)-complexes in the cleavage of phosphate diesters, systems resembling those metallo-nanoparticles, like micelles and vesicles, have been the subject of investigation since the mid-eighties of the last century. This paper reviews what has been done in the field and compares the different nanosystems highlighting the source of catalysis and frequent misconceptions found in the literature.

## 1. Introduction

Human beings are by nature emulative and scientists are not an exception to this behavior. Thus, it is not surprising that the recent hype about the nanoworld has spurred the renaming of old systems with new “nano” names. Micelles, aggregates of amphiphilic molecules and vesicles, spherical surfactant double-layers encapsulating a water pool segregated from the bulk solution, have been rebranded nanoparticles. The change of a name does not do much to a system but for the risk of the occasional “rediscovery” and “reclassification” under a new label of apparently new phenomena which, in reality, have been already studied and thoroughly investigated by others long ago. What we try to do in this review is to readdress relatively old research in metallomicellar and metallovesicular catalysis and compare it with what has been more recently reported on these systems and on a related one, i.e., nanoparticles passivated with a monolayer functionalized with metal complexes. Similarities and differences between the different colloids will be analyzed. We will focus on hydrolytic processes both for our familiarity with these reactions and because they are an excellent testing ground to prove similarities and differences in mechanism as well as relative efficiencies. They all share the property of being multivalent and, as far as catalysis is concerned, showing enzyme-like kinetic profiles.

Hydrolytic enzymes may be divided into two broad classes: those presenting a metal ion in their catalytic site and those devoid of it. To the last class belong mostly proteases, while to the first ones belong phosphate-cleaving enzymes. This is because a neutral amide bond is a much easier target for hydrolytic cleavage than an anionic phosphodiester of a nucleic acid. In facts, a metal ion provides the key ability to dissipate the ionic charge present on a substrate thus facilitating the attack by a similarly anionic nucleophile. Nevertheless, metal ions are also present in proteases for the important kinetic contribution they provide. Examples are constituted by carboxypeptidases that use the Lewis acidity of metal ions, typically Zn(II), to increase the electrophilicity of the carbonyl carbon and/or increase the nucleophilicity of water by stabilizing hydroxide in close proximity to the substrate. Similarly, enzymes that catalyze the hydrolysis of phosphate ester bonds use the functional groups of amino acids in a concerted fashion with the Lewis acidity of metal ions. One well-studied example is staphylococcal nuclease, which uses the electrostatic properties of a protein-bound Ca(II) ion and general base catalysis to hydrolyze DNA.

## 2. Surfactant-Based Hydrolytic “Metallonanosystems”

It should not be surprising that micellar, vesicular and, more recently, nanoparticles catalysis has used metal-catalyzed hydrolysis as a proving ground to test the enzyme-like properties of such systems [1,2,3]. All these nanosystems share two very attracting properties for a chemist, which are that of forming spontaneously once the proper monomeric constituents have been prepared and to confine several functional, and possibly reactive groups, in a small space. Accordingly, synthetic efforts are confined to the preparations of small, relatively simple molecules at variance with complex macromolecular model of metalloenzymes [4,5].

### 2.1. Metallomicelles

**Figure 1:**
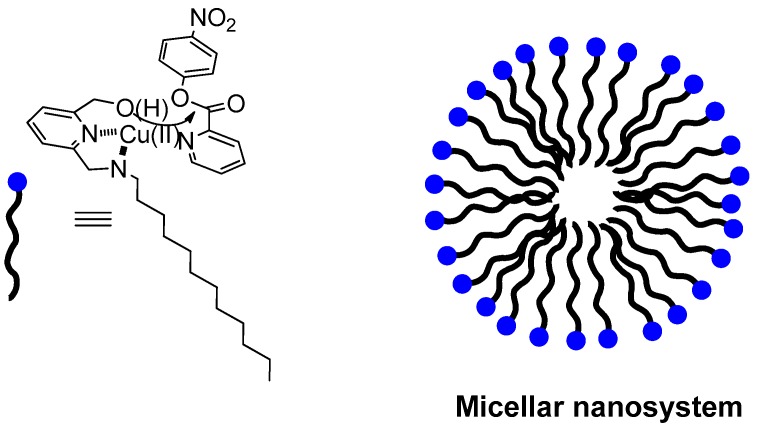
Micellar aggregates of amphiphilic Cu(II) complexes are particularly effective in the cleavage of ester substrates able to coordinate to the metal center. The built-in nucleophile of the ligand attacks the substrate carbonyl in a pseudo intramolecular process. The substrate depicted is *p*-nitrophenyl picolinate (PNPP) while the cartoon represents the nanometric micellar aggregate. The blue dot in the cartoon rendition represents the polar, metal-binding portion of the amphiphilic molecule while the black wave line represents the hydrophobic, hydrocarbon moiety.

Aggregates of amphiphilic molecules endowed with the ability to complex metal ions have been dubbed metallomicelles [6]. Micelles are spherical nanosystems roughly 3 nm in size. In an aqueous solution they expose the hydrophilic headgroups to the bulk water while the hydrophobic portion of each molecule is mostly buried in the interior. This is, however, an over approximation as it is well accepted that water molecules manage to permeate in the interior so that the “hydrophobic” environment is not much different from that of an alcoholic solution. In metallomicelles the polar headgroup is constituted by a metal complex. An early example was reported by Moroi et al. [7] who prepared poorly soluble Cu(II) alkylsulfonates. More or less at the same time, a bunch of laboratories started studying aggregates of metalloamphiphiles. Thus, Gutsche and Mei [8] found that the copper complex of a tetradentate ligand bearing a hydrocarbon chain catalyzed the hydrolysis of acetyl phosphate by a factor of ten. Catalytically active copper bolaforms were reported by Tonellato et al. [9] Several groups, including those of Tagaki [10] and Breslow [11] examined micelles containing heavy metals other than copper. In 1987 Menger [12], in response to the pressing quest for destroying nerve agents, i.e., phosphate-based chemical weapons, reported a striking example of an amphiphilic Cu(II)-complex very effective in the cleavage of phosphate triesters including some of these noxious chemicals. We argued that a built-in nucleophile would perform better than one recruited from the solvent. For this reason we prepared hydroxy-functionalized lipophilic pyridine-based ligands turning into surfactants upon metal ion complexation, and hence able to form micelles in aqueous solution [13]. The aggregates were particularly active catalysts in the cleavage of α-amino acid esters, i.e., substrates able to interact with the metal ion via coordination of the amino group. Most of the studies have been done with unnatural *p*-nitrophenyl picolinate (PNPP, Figure 1).

**Figure 2:**
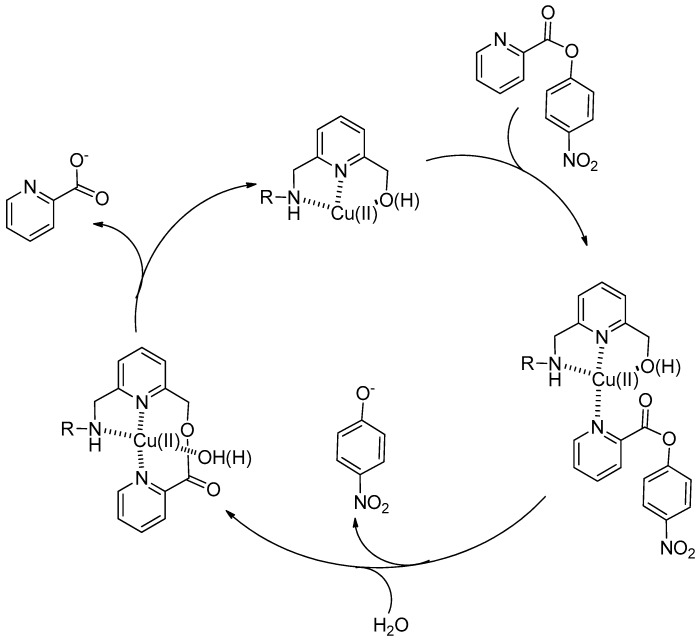
Catalytic cycle for the cleavage of a coordinating ester substrate by a metallo catalyst. First the substrate (in this case PNPP) coordinates to the catalyst with a built-in –OH nucleophile. The subsequent step represents the acylation of the catalyst (and its consequent inactivation) while in the last one the catalyst turns over after hydrolysis and the catalytic cycle may start again.

Typically, assessment of the catalytic efficiency of micellar (and vesicular) systems is performed using excess catalyst over substrate thus reversing the conditions used for enzyme catalysis. There are several reasons for that: (a) micelles existence implies a threshold concentration (critical micelle concentration, cmc, or critical aggregation concentration, cac) below which they fall apart into the monomeric surfactants; (b) excess substrate may alter the micellar structure; (c) in the presence of a built-in nucleophile a relatively stable, catalytically inactive (or very little active) intermediate could form. This leads to catalyst inactivation due to the lack of turn-over, an important property of natural enzymes. Nevertheless, and with these limitations in mind, pseudo-first-order rate constants obtained were up to a million-fold larger than those observed in pure buffer. A mechanism like that depicted in Figure 2 was suggested in which the deacylation of the intermediate is the rate determining step. If it is fast enough to ensure a fast turnover rate, then the system behaves as a true catalyst.

This was the case for the systems studied by Bhattacharya et al. based on 4,4-(dialkylamino)-pyridine (DAAP)-based ligand amphiphiles [14]. Catalytic turnover in the hydrolysis of *p*-nitro-phenyl hexanoate (PNPH) and *p*-nitrophenyl diphenyl phosphate (PNPDPP) was demonstrated in a CTAB comicellar medium with two equivalents of Cu(II). Similar tetradentate ligands showed the same behavior but in this case the active Cu(II)-complex had a 1:1 stoichiometry [15]. Work by the groups of Tagaki [16,17] and Engbersen [18] has further supported the catalytic relevance of a built-in nucleophile.

Apart from the clear kinetic advantage micellar aggregates provide with respect to monomeric catalysts, the real question is: what is the source of the rate acceleration? Do micellar aggregates provide something different and new in the catalytic process which can account for the observed rate accelerations? The result of our investigation on this point was that “in hydrolyses catalyzed nucleophilically, reactivities of micellized metal complexes do not differ significantly from those of monomeric catalysts in water” [19]. Therefore, the mechanism suggested in Figure 2 applies equally to the reaction in micellar aggregate as well as in bulk solution and no differences are generally observed also in the case of ligands with different structures or bearing nucleophiles different than the hydroxyl group [20,21]. Two relevant effects contribute to altering the reaction rate in these aggregates: (a) the pH, which is higher at the reaction loci because the interfacial cationic metal ions bring in not only counterions but also OH^−^; (b) increased concentrations of the reacting species (the nucleophile and the substrate) which are confined in the small volume of the interfacial region of the aggregates where the reaction occurs. Many examples of metallomicellar catalysts for the cleavage of activated carboxylic [22,23,24,25] and phosphoric [26,27,28,29] esters are in full accord with this interpretation. Remarkable exceptions have been, however, reported and they show that, in selected cases, even subtle changes in the medium at the reaction site are responsible for significant modifications of the mechanism of the reaction. This is not much different from what is observed in enzyme catalysis.

**Figure 3:**
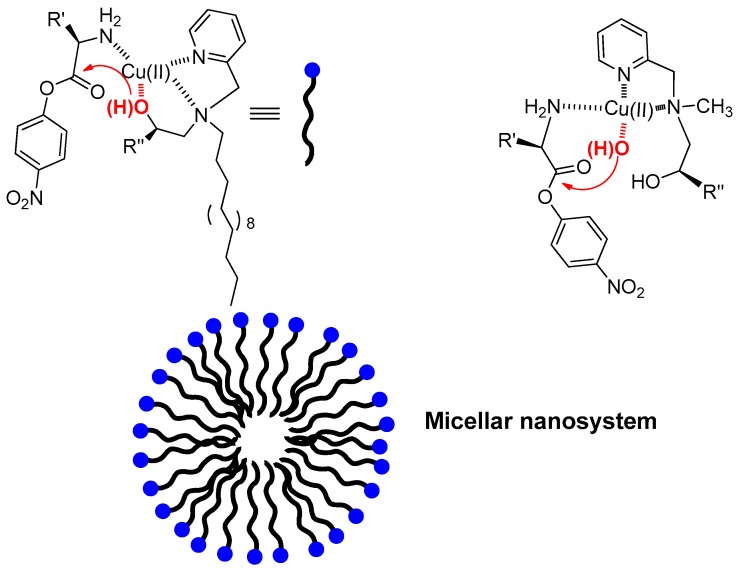
Enantioselective metallomicellar system and its corresponding monomeric catalyst (**right**). In the micellar environment the built-in nucleophile is involved in the coordination of Cu(II) and is the actual nucleophile leading to very fast pseudo intramolecular attack of the amino acid ester with high enantioselectivity. In the monomeric system fully solvated by water the OH-bearing arm of the ligand is replaced by a water molecule acting as the nucleophile. This leads to lower reactivity and negligible enantioselectivity. The blue dot in the cartoon rendition represents the polar, metal-binding portion of the amphiphilic molecule while the black wave line represents the hydrophobic, hydrocarbon moiety.

One of such examples was reported by us on studying the system depicted in Figure 3 [30,31]. It is not much different from those reported above but for the presence of a chiral center at the carbon holding the built-in nucleophile. Indeed, our idea was that of using it for the enantioselective hydrolysis of chiral amino acid esters. The Cu(II) complex of the most lipophilic ligand forms micelles that showed remarkable rate acceleration and enantioselectivity (k_S_/k_R_ ca. 10) in the cleavage of the *p*-nitrophenyl ester of phenylalanine (PhePNP). Strikingly, however, control experiments with a Cu(II) complex unable to form aggregates, not only showed very poor enantioselectivity but turned out to be an inhibitor of the cleavage process. We showed that the enantioselectivity with the micelle-forming ligand was strictly related to the formation of a ternary complex (ligand/Cu(II)/ester) as indicated by the almost complete absence of selectivity (k_R_/k_S_ = 1.5) when Z-protected phenylalanine (Z-PhePNP) was used as substrate. This and other experiments led to the conclusion that the observed behavior was correlated to the environment-controlled shift of the equilibrium of coordination of the ligand alcoholic arm to the metal center. In the case of the non-micellized complex water competition prevented the ligand hydroxyl from the coordination to the metal. The reactivity was hence decreased as the built-in nucleophile was no longer available and the ligand chirality had poor effect on the reaction. By the contrary, when the ligand hydroxyl was bound to the metal ion in the less polar micellar environment the system was able to take advantage of the pseudo-intramolecular attack to the coordinated ester. This modulation of the coordination sphere of the metal ion in aggregates appears to be a general phenomenon and has been observed also with other lipophilic metal complexes [32]. This fine tuning of the coordination sphere of the metal ion highlights how much sensitive it is even to very small changes of the polarity of the environment. Noteworthy, the modification of the medium at the reaction site affects reactivity also on the surface of monolayer-protected gold nanoparticles, although, in these cases it occurs in systems devoid of the metal ion as a catalytic unit [33,34].

It is worth mentioning here that other groups have also studied enantioselective catalysis using chiral metallomicelles. At variance with the micellar system discussed above, most of these studies have been performed with substrates unable to coordinate directly to the metal center. In these cases, a tight substrate-micelle interaction was ensured by the lipophilicity of the substrate. Thus, Engbersen [35] prepared a family of enantiopure lipophilic ligands based on 1,10-phenanthroline and investigated the cleavage of chiral *N*-protected amino acids esters in the presence of different surfactants and metal ions (Zn(II), Co(II), Cu(II), and more). The highest enantioselectivity reported (k_R_/k_S_ ratio of ca. 15) was with the Co(II) complex. Very intriguing was the observation of relevant changes in enantioselectivity by using mixtures of different surfactants and by varying the lipophilicity of the substrate. This was likely due to the inability of the substrate to bind to the metal center and, hence, the control of the binding geometry within the catalytic site was mostly governed by lipophilicity. A similar conclusion can be drawn also by analyzing the work of You and coworkers [36,37,38]. In fact, also with their systems the activity depends on several factors (apart from the structure of the ligand and the type of metal ion) that include the lipophilicity of the substrate and the surfactant used as comicellizing agent.

### 2.2. Metallovesicles

Metallovesicles (or metalloliposomes) are nanostructures formed by surfactants, typically bearing two hydrocarbon chains connected to the hydrophilic portion of the molecules, that aggregate forming a spherical bilayer encapsulating a water pool at its interior. Compared to micelles, these are bigger aggregates (20–100 nm) characterized by a tighter packing of the monomers. Indeed, the packing requirements of a surfactant in the vesicle bilayer are much more stringent than in a micelle and, as a consequence, the “order” of the aggregate is higher than the “order” of the micelle. This is true especially at a temperature below the phase transition temperature (Tc), when the membrane is in the gel state. Above this temperature, kinks occur in the hydrocarbon chains making the packing of the surfactants less efficient. Nevertheless, reactivity in these systems is not much different from that in micelles described in the previous section [39]. The use of lipophilic molecules functionalized with dinuclear Zn(II) complexes proved to be the most successful approach, indicating that a dinuclear catalytic site is, likely, the most active reaction site [39,40]. Rate accelerations are often spectacular and the systems show also the ability to cleave plasmid DNA and single stranded DNA. Within the vesicular membrane the metallocatalytic site may also take advantage of the presence of additional functional groups similar to those typically present in enzymes [40]. In the case of lanthanide ions even a very low binding to the surface of a vesicular aggregate devoid of a classical ligand moiety was able to induce significant rate acceleration in the cleavage of phosphates [41]. Such an effect was ascribed to the formation of bimetallic reactions sites promoted by the accumulation of lanthanide ions on the liposome surface.

**Figure 4:**
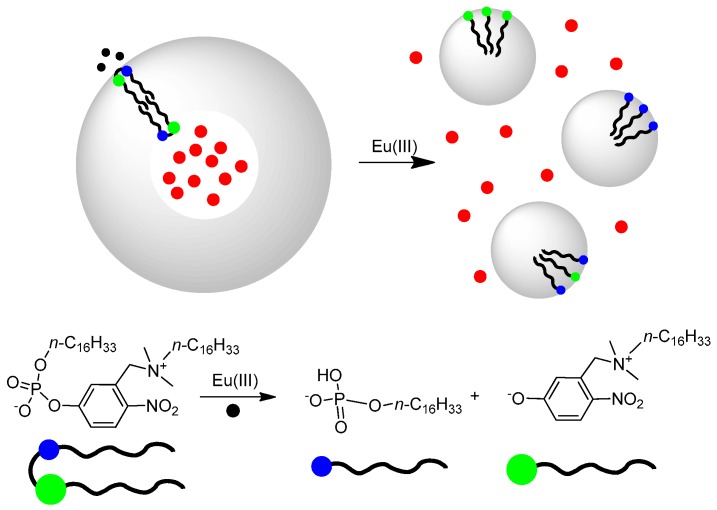
Vesicles formed by the zwitterionic, phosphate-based amphiphile depicted at the bottom interact with Eu(III) ions that accelerate the cleavage of the phosphate (bottom equation) leading to amphiphilic molecules forming micelles. This leads to the release of the dye (carboxyfluorescein, red dots) trapped in the internal water pool of the vesicles. The blue dot in the cartoon rendition represents the ammonium portion of the amphiphilic molecule while the green one represents the phosphate headgroup. Black wave lines represent the hydrophobic, hydrocarbon moieties.

The existence of two aqueous regions, the bulk and the internal pool to which the outer and inner leaflet of the bilayer are exposed, respectively, may however, lead to altered reactivity. In fact, only fast permeation of the metal ions and/or the substrates across the bilayer allows to take advantage of all available catalytic sites while slow permeation may prevent the availability of the catalytic units present in the interior of the aggregate. A further problem is connected to the movement of the surfactants from one leaflet to the other (referred to as ‘flip-flop’). A fast flip-flop compared to the reaction studied will allow taking advantage of all catalysts present even under conditions of slow permeation across the bilayer. Moss et al. have achieved chemical differentiation between the internal and external surfaces of a vesicle through selective esterolysis [42,43]. These authors have also shown how the flip-flop of the surfactant is dramatically dependent on the structure of the lipophilic backbone [44,45]. We have shown that all these parameters indeed modulate the reactivity of hydrolytic metallovesicles [46]. With surfactants bearing a substrate amenable to esterolytic cleavage by a metal ion it was possible to cleave selectively the external monolayer while leaving intact the internal one and monitor in real time the flip-flop process [47,48]. In cases in which the cleavage of the surfactant results in the removal of one of the lipophilic chains, vesicles turn into micelles leading to a ‘decapsulation’ process, i.e., the release of any substance trapped in the internal water pool (Figure 4) [49]. A very peculiar case of shift from micelles to vesicles and vice versa was recently reported by us using metallosurfactants as building blocks [50].

A case in which being a micelle or a vesicle does make the difference is enantioselective hydrolysis. In view of the higher degree of organization of vesicles respect to micelles mentioned above one could expect better results in term of enantioselectivity. When the lipophilic catalyst reported in Figure 3, discussed in the previous section, was embedded in a vesicular bilayer a three-fold increase in enantioselectivity was observed [51].

**Figure 5:**
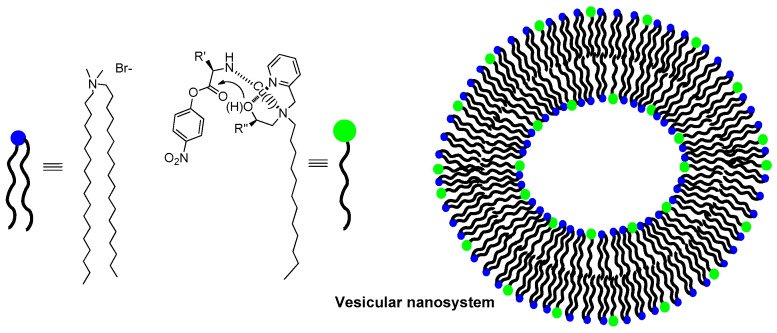
Cartoon representation of the vesicles made up of the amphiphilic, chiral Zn(II) (*S*-enantiomer) complex shown in Figure 3 embedded in a *N,N*-dimethyl-*N,N*-di-*n*-octadecyl ammonium bromide matrix. The cartoons represents the situation below the phase transition temperature. The less ordered membrane formed above the phase transition affects dramatically the rate of cleavage of the two enantiomers of the *p*-nitrophenylester of phenylalanine. In particular, it slows down the reactivity of the *R*-enantiomer thus decreasing the enantioselectivity of the process. The blue dot in the cartoon rendition represents the ammonium headgroup of the amphiphilic molecule while the green one represents the metal complex headgroup. Black wave lines represent the hydrophobic, hydrocarbon moieties.

A hint of the source of increased enantioselectivty was the Arrhenius profile measured for the reaction of the two enantiomers of the substrate. The enantioselectivity was higher at low temperature and decreased sharply at temperatures near and above the phase transition of the membrane. Interestingly, this decrease was associated with the slowing down of the reaction of the faster enantiomer, the slower one being almost unaffected (Figure 5). This has been explained with enantioselectivity arising from the compartmentalization of the two reacting ternary complexes in different locations of the aggregate. The increase in temperature above the phase transition of the bilayer would be associated with an increase of its fluidity and, consequently, the segregation of the different species would become less efficient. When the faster enantiomer moves into a more hydrated region, the mechanism of the reaction changes and the overall reaction rate, counterintuitively, becomes slower in spite of the temperature increase.

## 3. Metallonanoparticles

Clusters of gold atoms (2–100 nm in size) covered by a monolayer of organic molecules constitute an interesting example of self-assembled systems [52,53]. Typically, individual molecules are anchored on the gold surface via a thiol that ensures a strong interaction with the metal. Depending on the nature of this interaction it may range from 18 (donor-acceptor bond) to 48 kcal/mol (covalent bond) [54]. This means that these thiols are kinetically inert (i.e., their exchange rate with those in solution is slow) at least on the time scale of most catalyzed reactions. Although the monolayer covering a gold nanoparticle may be regarded as very similar to a surfactant aggregate (either a micelle or vesicle), the rate of the exchange of the monomers makes for a huge difference between the two classes of systems. One may consider micelles and vesicles as dynamic systems as the constituent monomers are in (relatively fast) equilibrium with the aggregates while the gold nanoparticles (AuNPs) are static ones. Furthermore, thiols anchored on the surface of a cluster of gold atoms, present a unidirectional orientation of these molecules, which means that the self-assembly process contains an element of topological control, absent in micelles and vesicles. The monolayer can play a crucial role in determining the catalytic properties of the system. This may occur either through general effects (pH, polarity) as in micelles and vesicles or, by embedding the catalyst within the monolayer rather than on the periphery. Such a situation could create an enzyme-like catalytic cleft which may affect substrate orientation and thus the selectivity of product formation. This latter situation cannot be achieved with micelles and vesicles because, contrary to AuNPs, the orientation and arrangement of the surfactants in the aggregate can be very little controlled by the scientist.

**Figure 6:**
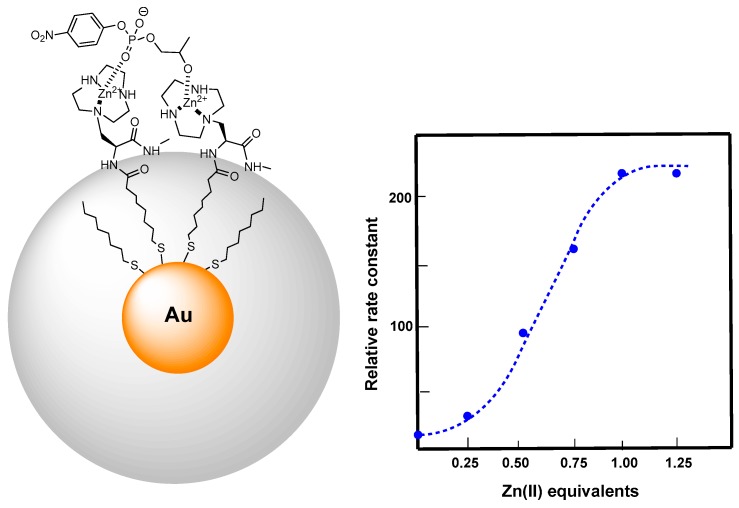
Triazacyclononane-functionalized thiols were used to prepare AuNPs with a mixed monolayer (**left**); Upon complexation with Zn(II) the AuNPs turn into powerful catalysts for the cleavage of HPNPP. The rate acceleration measured when continuously adding Zn(II) ions up to saturation shows a sigmoidal behavior (**right**) hinting to cooperation between two metallic centers in the catalytic process.

An early example addressing phosphate cleavage with AuNPs was reported by us by using a thiol bearing a triazacyclononane-functionalized amino acid [55] grafted on a gold cluster in a 1:1 mixture with octanethiol [56]. By studying the cleavage of the RNA-model substrate 2-hydroxypropyl-*p*-nitrophenyl phosphate (HPNPP), working at a fixed concentration of nanoparticles and progressively adding Zn(II) ions, we found a sigmoidal reactivity profile (Figure 6). This curve was used to support a cooperative mechanism involving at least two metal ions. The Michaelis-Menten-like profile of the kinetic reaction led us to coin the definition of “nanozymes” for these systems.

The nanoparticles were also active with dinucleotides, UpU in particular. The desolvation of the substrate from the bulk aqueous solution proved an important factor in enhancing the activity of the AuNP-based catalyst. By studying a series of nanoparticles passivated with Zn(II) complexes of increased hydrophobicity we found the cleavage of HPNPP was accelerated by a further order of magnitude just by changing the medium polarity. 

This led us to conclude: “the results [...] provide an insightful picture of the potential of monolayer-coated nanoparticles as enzyme mimetic systems. On one hand they allow the self-assembly of the bimetallic catalytic site, while on the other, the simple modification of the structure of the coating thiol by the insertion of longer alkyl chains leads to the obtainment of a microenvironment with a polarity lower than that of bulk water. In such conditions, the ability of the metal ions to stabilize, likely by electrostatic interaction (coordination), the dianionic transition state formed during the HPNPP cleavage is increased, and consequently, the reaction is faster. Indeed, to the best of our knowledge, k_cat_ values for its cleavage corresponding to t_1/2_ less than 20 s, like those we observe with (the best performing AuNP), have never been reported previously for Zn(II)-based catalysts working in water” [57].

**Figure 7:**
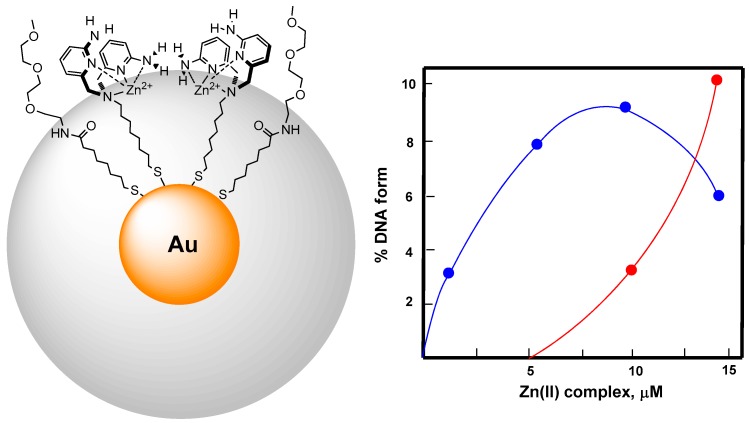
AuNPs functionalized with a bis-(2-amino-pyridinyl-6-methyl)amine (BAPA) functionalized thiol (**left**) proved extremely effective in cleaving plasmid DNA; By following the early stages of the cleavage process (**right**) we found that double strand cleavage was much higher than expected. In fact the amount of circular form (blue dots) formed as a consequence of a single strand cleavage is very quickly surpassed by the linear one (red dots), consequence of a two-strand cleavage.

HPNPP, as a model of RNA, has the advantage of containing an hydroxyl group that, acting as intramolecular nucleophile, greatly increases the cleavage rate of the phosphate bond. This is why RNA is cleaved much faster than DNA, too. In fact, a phosphate diester devoid of such an intramolecular nucleophile requires, for its cleavage, a more efficient catalyst. With this in mind, we functionalized the monolayer of AuNPs with a ligand in which the action of the metal ion could be assisted by ancillary groups providing extra contribution to the catalysis. We prepared a bis-(2-amino-pyridinyl-6-methyl)amine (BAPA) functionalized thiol (Figure 7), as the BAPA-Zn(II) complex is known to be able to elicit the cooperation between metal Lewis acid activation and hydrogen-bonding to achieve increased hydrolytic activity toward phosphate diesters [58,59,60,61,62,63,64,65]. The nanoparticles were prepared following a two-step protocol [66] relying on the preliminary passivation of the gold surface with a secondary amine followed by the final introduction of the functionalized thiol. The resulting Zn(II) nanoparticles proved to be one of the most effective catalysts reported so far for the cleavage of the DNA model phosphate, bis-*p*-nitrophenyl phosphate (BNPP) [67]. The second-order rate constant observed was not only 100-fold better than that of the monomeric complex but more than 60,000 higher than that of the OH–catalyzed background reaction. The dependence of the rate constants on the equivalents of Zn(II) added showed the occurrence of two catalytically relevant situations. At low Zn(II) loading, a mononuclear complex was the active species while at Zn(II) saturation, a dinuclear complex was the catalyst. This represents a significant difference from the previous examples with HPNPP, where only the dinuclear catalysts were significantly effective. When we challenged our AuNPs in the cleavage of plasmid DNA we were rewarded by very interesting results. The hydrolytic process with this substrate can be conveniently followed by gel electrophoresis. The conversion of the compact supercoiled conformation of the polymer (form I) to the relaxed circular one (form II) following a single cut in one of the two strands is associated with a remarkable change in mobility. If the following cut occurs in the other strand in close proximity (less than 12 nucleotides far) to the first one, a further change occurs with the linearization of the double strand (form III), also easily detectable in the gel. Statistically only one cut over 100 occurs in the proper position to lead to linearization. Consequently, linear form starts to appear in significant amount only after most of the supercoiled has been cleaved. Strikingly, in the presence of the nanoparticles linear DNA started forming after only 10% of the original supercoiled DNA was cleaved and at 16% cleavage it became the most abundant form present (Figure 7, right). This clearly indicated that the nanoparticle was able to perform multiple simultaneous cuts on the double strand taking advantage of multivalency on two counts: first by creating the dinuclear catalytic site and second by providing on the same AuNP multiple catalytic (dinuclear) sites. This latter property led to the efficient linearization of the supercoiled DNA, a behavior common in enzymes but never observed with other artificial catalysts.

Catalytic systems could also be obtained by using lanthanide-complexes. AuNPs coated with Ce(IV) chelating groups were prepared by us [68]. They proved to be highly active in promoting the hydrolytic cleavage of HPNPP. A 2.5 million-fold rate acceleration at 120 mM (pH 7, 25 °C) of Ce(IV) was observed, compared to the uncatalyzed reaction. This corresponds to a reduction of the substrate half-life time from 2000 years to a few hours. The AuNP-Ce(IV) system was up to 2 orders of magnitude more reactive than any mono- and binuclear Ce(IV) complex reported. Also in this case, the source of such reactivity has been ascribed to the cooperativity between the self-organized metal ions on the surface of the nanoparticles. Uncomplexed Ce(IV) is, however, more active, in sharp contrast with the results reported above with Zn(II)-based nanoparticles where free Zn(II) is far less active. The explanation is that only Ce(IV) and not Zn(II) forms oligomeric (active) clusters in solution [69].

**Figure 8:**
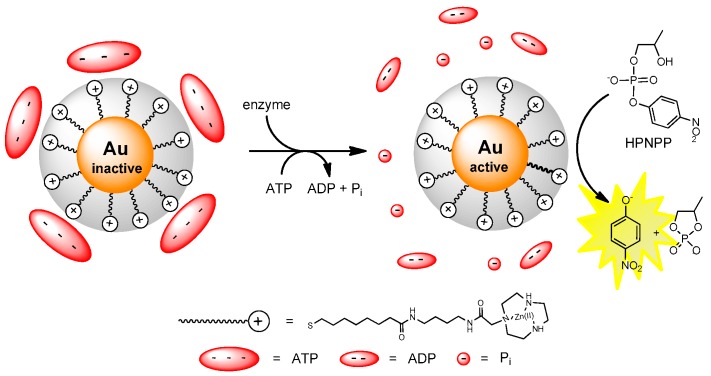
Catalytic cycle leading to enzyme detection characterized by kinetic signal amplification. When ATP binds to the triazacyclononane-Zn(II) functionalized AuNP it inhibits HPNPP binding and cleavage. When the enzyme cleaves ATP it no longer binds to the AuNP surface leaving room for HPNPP binding. As a consequence HPNPP is cleaved releasing *p*-nitrophenol used as a tool to quantify the amount of enzyme present.

Not surprisingly, cationic nanoparticles show a very high affinity for oligoanions. This includes anionic oligopeptides, ATP, ADP, oligophosphates, and anionic proteins [70]. In line with what is expected for electrostatically-driven interactions, a systematic study of a series of phosphates and carboxylates revealed that the affinity of the oligoanions for the catalytic monolayer surface was strictly related to the number of negative charges of the probe. The strength of the interaction was dictated by the nature of the cationic group on the surface of the AuNP: ammonium groups proved less efficient than cationic metal complexes. This property was used for the detection of enzyme activity taking advantage of the catalytic properties of the monolayer of metallonanoparticles [71]. The enzyme assay (Figure 8) relied on the use of an anionic peptide acting both as the enzyme substrate and as a species strongly binding to the surface of the cationic AuNPs. The set up took advantage of the fact that the enzyme substrate and HPNPP (both anionic) were competing for the surface of the nanoparticle. With the polyanionic peptide showing an higher affinity for the Zn(II) AuNP, HPNPP was prevented from interacting with the nanoparticle and hence was not cleaved by the nanosystem. However, upon hydrolysis by the enzyme, the peptide (acting as an inhibitor) was fragmented loosing affinity for the monolayer surface. As a consequence, catalytic activity against HPNPP of the monolayer was restored, resulting in the production of the *p*-nitrophenolate anion as a reporter molecule of the activity of the enzyme. An attractive feature of the system was the catalytic signal amplification [72] so that the amount of produced signal was connected to the turn-over number of the catalyst.

**Figure 9:**
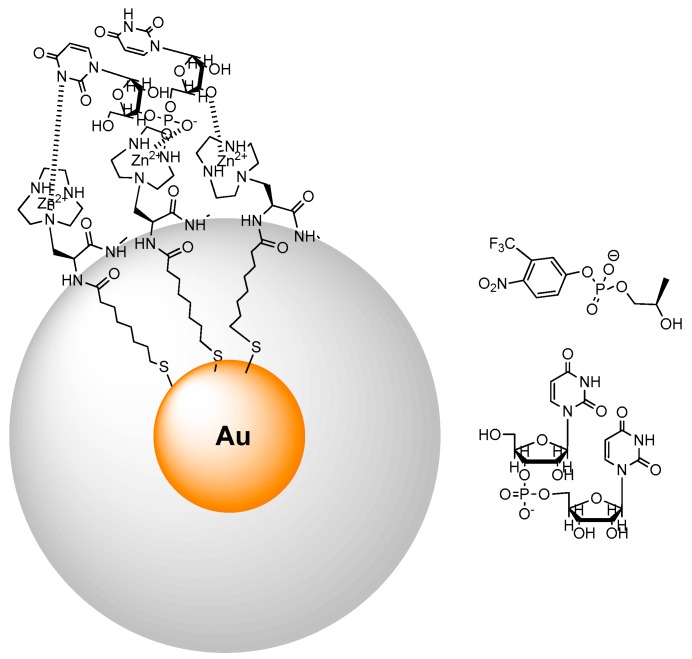
A chiral thiol forming the passivating monolayer of AuNP (**left**) provides the chiral environment for the enantioselective cleavage of chiral posphate diesters like those depicted on the right.

We have shown above, for metallomicelles and metallovesicles, that chiral discrimination in the hydrolytic cleavage is feasible. Recently we demonstrated for the first time [73] enantioselectivity in RNA cleavage by a synthetic metallonanoparticle. Thiols containing chiral Zn(II)-binding head groups have been self-assembled on the surface of gold nanoparticles (Figure 9). This resulted in the spontaneous formation of chiral bimetallic catalytic sites that displayed different activities (kcat) towards the enantiomers of an RNA model substrate similar to HPNPP. Substrate selectivity was observed when the nanozyme was applied to the cleavage of the dinucleotides UpU, GpG, ApA, and CpC, and remarkable differences in reactivity were observed for the cleavage of the enantiomerically pure dinucleotide UpU.

The better enantioselectivity observed with UpU with respect to the HPNPP-like substrate was attributed to the ability of the dinucleotide to provide an extra binding site to a metal via the uracil of the nucleobase uridine. This would translate into a higher energy difference in the transition state. It is worth mentioning that the unassembled catalytic units did not induce any enantioselectivity. Hence the nanostructure of the nanozyme provides the unique chiral environment allowing the differentiation of the two diastereomeric transition states.

## 4. Comparison between the Nanosystems and the Quest for Cooperativity

Obviously one is curious to know how the different metallonanocatalysts perform not only in comparison between themselves but also with respect to other systems. We will focus on available data (mostly from our own labs) considering only systems based on triazacyclononane as the ligand, Zn(II) as the metal ion, and HPNPP as the substrate. This doesn’t imply that the triazacyclononane-Zn(II) complex is the best one for catalysis but it is the one for which more data are available. We have summarized a few numbers in Table 1.

Analysis of the data reported in Table 1 reveals that gold nanoparticles-based systems are far superior to any other catalyst (micellar or monomolecular, like dendrimers or tripodal molecules). Several reasons contribute to this: (a) the possibility to better control the polarity of the solvent [57] as a lower solvation of the nucleophile increases its nucleophilicity; (b) a decreased mobility of the catalyst at the catalytic site due to the packing of the molecules on the monolayer covering the gold cluster; (c) local effects (experienced also by micelles and vesicles) the most notable one being the increase of the pH in the catalytic region [78].

We and others have demonstrated that, with triazacyclononane-Zn(II)-based catalysts, a dinuclear catalytic site is far more effective than a mononuclear one [79,80]. Indeed, with all our AuNPs, plots of the catalytic efficiency against the equivalents of Zn(II) added results in sigmoidal curves. The sigmoidal inflection was interpreted with the attainment, within the monolayer, of the critical condition that allows two Zn(II) complexes to operate in a concerted, cooperative way. Preliminary data not yet published from our own laboratories indicate that a similar behavior may also be present in micelles and, in this case, catalytic effectiveness comparable with that of nanoparticles is reached. Hence the most relevant contribution of the nanoparticles is their ability to promote the collaboration of two metal ions to form an effective bimetallic reaction site. Interestingly, a detailed comparison with molecular bimetallic complexes revealed that bimetallic sites on the nanoparticles are characterized by substantially higher affinity for the substrate, while the intrinsic reactivity is not or little affected.

**Figure 11:**
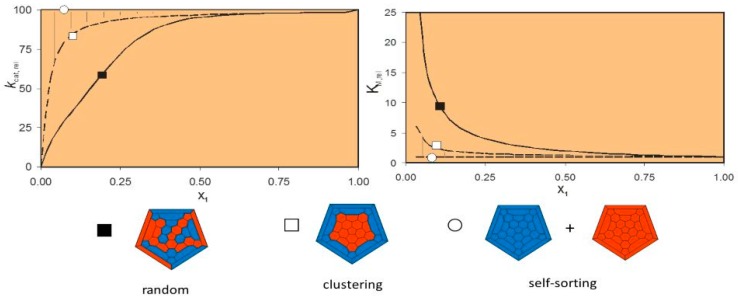
Calculated kinetic (k_cat_, **left panel**) and binding (K_m_, **right panel**) profiles for the cleavage of HPNPP by AuNPs with a mixed monolayer comprising a catalytic triazacyclononane-Zn(II) thiol and an inactive one (featuring a oligoether functional group) as a function of the mole fraction. The curves simulate the situation with thiols distribution ranging from random (filled squares), cluster formation (empty squares) and total sorting (empty circles). They assume that once all available Zn(II) complexes are able to form a dinuclear catalytic site the reaction rate doesn’t increase any further. The tiles representation on the bottom gives an idea of the thiols arrangement on the surface for the three different situations (blue and red are the inactive and active thiols, respectively). The experimental data fit nicely the unsorted thiols arrangement and confirm the reaction rate doesn’t increase once the dinuclear catalytic sites are formed (ca. 0.4 mole fraction, **left panel**). The dissociation constant, however, decreases smoothly till 100% reactive thiol covers the AuNP surface (**right panel**).

Such conclusions are supported by more detailed investigations aimed at understanding at what loading the catalytic site is set and whether further metal centers or new specific parameters could be beneficial to the catalytic transformation. We have verified [77] that the multivalency of the system causes an increase of the number of potential dimeric catalytic sites as a function of the mole fraction of ligand in a AuNP coated with a mixed monolayer comprising also an inert (polyether) component. This is obvious because as the metal complexes get closer dinuclear catalytic centers start to form. This causes the mentioned increase in binding affinity (and a decrease in K_m_). As for the catalytic performance (measured as k_cat_), we observed that for values of the mole fraction of Zn(II) complex >0.4 (with respect to the inert additive), the apparent k_cat_ remained practically constant. This implies that above this threshold value of mole fraction all possible dinuclear catalytic sites are formed (Figure 11). However, since the apparent K_m_ value showed an exponential decay as a function of the surface loading, this resulted in a continuous increase in k_cat_/K_m_ as a function of the catalyst loading. Without a proper analysis, this would have been ascribed to an improvement in the catalytic site performance or to other reasons of chemical origin (the ‘dendritic’ effect). The experimental evidence clearly indicate that the catalytic site is more or less the same once the dinuclear couple is formed and there is no catalytic advantage for the proximity of further metal complexes.

## 5. Conclusions

In this short review we have shown how metallocolloids, i.e., nanometer size particles dispersed in solution (like micelles, vesicles and gold nanoparticles) able to bind metal ions may behave like powerful enzyme mimetic agents in hydrolytic reactions: in a word, like hydrolytic “nanozymes” [81]. Hydrolytic nanosystems are, obviously, not confined to the colloids we have described here [82,83]. Interesting examples are constituted by dendrimers [76,84], synthetic polypeptides [85] and designed proteins [86]. With the systems described here over one million fold rate accelerations have been observed in the cleavage of selected substrates with respect to the uncatalyzed cleavage process. The source of catalysis was attributed to partial desolvation, concentrations effect, local pH changes and cooperativity between reacting centers, particularly in the formation of very active dinuclear catalytic sites. Only in very specific cases the nanosystems were able to alter significantly the coordination mode of the ligand to the metal ion leading to completely different reaction pathways. Also these changes, although characterized by a very impressive outcome, were attributed to changes of the solvation in the reaction loci, typically much less hydrated than that obtained in bulk water. The ability to perform enantioselective processes has been also associated with differential solvation in conjunction with the chiral environment provided by the nanosystem. There is still ample room for improvement as the rate accelerations and enantioslectivities provided by natural hydrolytic enzymes are still far better than those so far obtained with synthetic nanozymes.

The research is now moving into the exciting field of system chemistry were the aggregation modes of the constituent monomers can be controlled by proper design by the scientist. Indeed, one may conceive situations in which the aggregates may provide transient environments for the occurrence of reactions as a function of a temporary condition: very much the same of what happens in the biological world where reactions are turned on and off following the change of the conditions like the activation of enzymes or a particular situation in a membrane. Systems providing transient reaction sites controlled by external stimuli have been already reported [50]. We expect an enormous development in this field in the coming years.

## Figures and Tables

**Figure 10:**
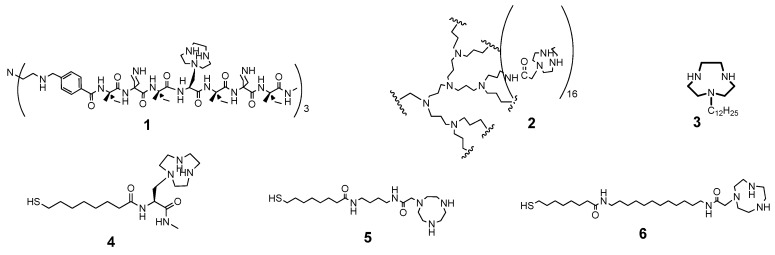
Structure of ligands reported in Table 1.

**Table 1:** Second order rate constants (k_2_ or k_cat_/K_m_) for several 1,4,7-triazacyclononane-Zn(II)-based nanometallocatalysts.

Ligand ^1^	System	k_2_ or k_cat_/K_m_ (s^−1^M^−1^) ^2^	Kinetic Advantage	References
1,4,7-Triazacyclononane	Monomer	0.007	1	[56,74]
**1**	Monomolecular trispeptide	0.033	5	[75]
**2**	Monomolecular dendrimer	0.64	91	[76]
**3**	Micelle	0.028	4	[56]
**4**	AuNP	4.4	629	[56]
**5**	AuNP	21.7	3100	[77]
**6**	AuNP	638	91,143	[57]

^1^ Ligand numbers refer to the structures depicted in Figure 10; ^2^ At pH = 7.4 and 40 °C.

## Abbreviations

The following abbreviations are used in this manuscript:

PNPP: *p*-Nitrophenyl picolinate
DAAP: 4,4-(Dialkylamino)pyridine
PNPH: *p*-Nitrophenylhexanoate
PNPDPP: *p*-Nitrophenyl diphenyl phosphate
CTAB: Cetyltrimethylammonium bromide
AuNP: Gold nanoparticle
HPNPP: 2-Hydroxypropyl-*p*-nitrophenyl phosphate
BAPA: Bis-(2-amino-pyridinyl-6-methyl)amine
BNPP: Bis-*p*-nitrophenyl phosphate
